# Novel compound heterozygous variants in *MARVELD2* causing autosomal recessive hearing loss in two Chinese families

**DOI:** 10.1002/mgg3.2502

**Published:** 2024-07-30

**Authors:** Xinyu Shi, Xiaozhou Liu, Yanjun Zong, Zhengdong Zhao, Yu Sun

**Affiliations:** ^1^ Hubei Province Key Laboratory of Oral and Maxillofacial Development and Regeneration Wuhan China; ^2^ Department of Otorhinolaryngology, Union Hospital, Tongji Medical College Huazhong University of Science and Technology Wuhan China; ^3^ Institute of Otorhinolaryngology, Union Hospital, Tongji Medical College Huazhong University of Science and Technology Wuhan China

**Keywords:** hearing loss, *MARVELD2*, targeted next‐generation sequencing, tricellulin

## Abstract

**Background:**

Hereditary hearing loss is an important component of congenital hearing loss. *MARVELD2* (OMIM ID:610572), located in the DFNB49 locus, which encodes a tight junction protein tricellulin playing an important role in the sensory epithelial barrier of the inner ear, may contribute to nonsyndromic autosomal recessive hereditary hearing loss.

**Methods:**

Two Han Chinese pedigrees with hearing loss underwent clinical and genetic analyses. Variants were detected by targeted next‐generation sequencing and sequencing data were compared with the Human Genome Reference (GRCh 37/hg 19) to identify mutant genes and loci. Furthermore, online tools such as RDDC, SpliceAI, and REVEL were used to predict risks from different variants.

**Results:**

Both two probands failed neonatal hearing screening and were diagnosed with sensorineural hearing loss. A total of 3 mutations were detected in the two families, c.1331+1G>A, c.1325A>G, and c.782G>A. According to ACMG/AMP guidelines, they were judged to be pathogenic, uncertain significance, and uncertain significance, respectively.

**Conclusions:**

These findings contribute to a better understanding of the relationship between different variants of *MARVELD2* and hearing. This could further expand the spectrum of deafness gene mutations and contribute to deafness patient management and genetic counseling.

## Graphical abstract

Targeted next‐generation sequencing was used to identify  novethreel variants of *MARVELD2*, and online tools were used to analyze variants' pathogenicity, which could further expand the spectrum of deafness gene mutations.

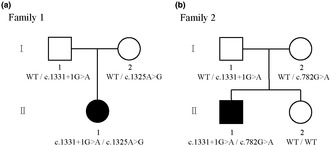

## 1 INTRODUCTION

Hearing loss presents a significant global public health concern with substantial social implications (Chadha et al., 2021). Universal newborn hearing screening (UNHS) has shown that the prevalence of permanent hearing loss is estimated at 1.1/1000 children (Butcher et al., 2019). This number is expected to increase with the rising adoption of newborn hearing screening protocols. Genetic factors account for 50%–60% of hearing loss cases in children in developed nations (Koffler et al., 2015). Currently, over 125 genes associated with non‐syndromic hearing loss have been identified (Walls et al., 2024), encoding proteins within the auditory pathway and leading to a wide range of manifestations. Non‐syndromic sensorineural hearing loss (SNHL) makes up about 70% of congenital hereditary deafness, with the most common genetic patterns being autosomal recessive inheritance (80%) and autosomal dominant inheritance (20%). Less than 2% of cases are X‐linked SNHL, and mitochondrial SNHL is less than 1% (Shearer et al., 1999).

In 2004, two closely related families were reported in which all affected individuals had congenital bifocal sensorineural hearing loss. Subsequently, linkage evidence was identified in the 5q12.3‐q14.1 region of the chromosome, and a new hereditary deafness locus DFNB49 was defined (Ramzan et al., 2005). The *MARVELD2* (OMIM ID: 610572) gene, also known as *TRIC*, is located on chromosome 5q13.2 and linked to the DFNB49 locus (Riazuddin et al., 2006). The *MARVELD2* gene encodes tight junction‐associated protein tricellulin. This transmembrane protein is mainly concentrated in the three‐cell tight junction (tTJ) of all tissue epithelial cells and plays an important role in maintaining the epithelial barrier (Ikenouchi et al., 2005). Tricellulin was observed in support cells, hair cells, and marginal cells of stria vascularis in the inner ear (Riazuddin et al., 2006). *Marveld2*‐knockout mice showed early rapid progressive hearing loss and hair cell degeneration was observed (Kamitani et al., 2015).

So far, only a few cases of hearing loss related to *MARVELD2* have been reported (Babanejad et al., 2012; Chishti et al., 2008; Mašindová et al., 2015; Nayak et al., 2015; Riazuddin et al., 2006; Sadeghi et al., 2020; Šafka Brožková et al., 2012; Taghipour‐Sheshdeh et al., 2019; Zheng et al., 2019). More cases of gene mutation need to be collected to understand its molecular mechanism. Here, we report two Chinese families with deafness caused by *MARVELD2* heterozygous variants, which may contribute to a better understanding of the relationship between tricellulin and phenotypes.

## 2 FAMILY DESCRIPTION AND METHODS

### 2.1 Ethical compliance

The study was approved by Medical Ethics Committee of Tongji Medical College, Huazhong University of Science and Technology (2022 S041). Before participation, all participants or their parents must give written initial informed consent per the Declaration of Helsinki.

### 2.2 Family description

**FIGURE 1:**
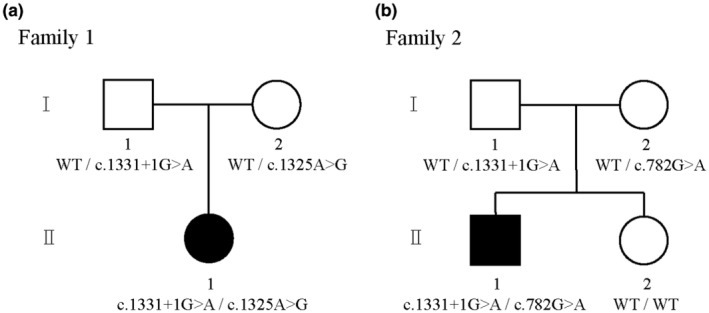
Family pedigrees of two probands. (a) In Family 1, proband II‐1 carries compound heterozygous variant c.1331+1G>A from her father and c.1325A>G from her mother of MARVELD2. (b) In Family 2, proband II‐1 carries compound heterozygous variant c.1331+1G>A from his father and c.782G>A from his mother of MARVELD2. Probands are marked in black. WT means wild type.

The two families reported in this paper are both Chinese Han families. The two probands failed neonatal hearing screening and were diagnosed with sensorineural hearing loss. Among them, the proband of family 1 is a 3‐year‐old girl, the proband of family 2 is a 9‐year‐old boy (Figure 1). In their pedigree, except for the proband, there was no deafness within three generations. Their mothers were screened to rule out TORCH (Toxplasma, rubellavirus, Cytomegalo virus, herpes simplex virus and other) infection and had no history of specific medications during pregnancy and perinatally.

### 2.3 Clinical examination

Both probands underwent audiological examinations such as the otoscopic examination, auditory immittance, otoacoustic Emissions (OAE), and auditory steady‐state–evoked responses (ASSR) or auditory brainstem response (ABR). Parents reported no history of miscarriage or stillbirth. No ear deformities or other systemic abnormalities were found in the probands. The physical examination, otoscopy, and medical history were performed at the outpatient clinic of Wuhan Union Medical College Hospital.

### 2.4 Variant detection and analysis

The method has been described in detail in our previous articles (Chen et al., 2020; Jin et al., 2022; Liu et al., 2021). We sampled peripheral blood from the probands and their parents for high‐throughput sequencing of 218 genes (Supplementary 1), including *GJB2*; *SLC26A4*; *MT‐RNR1* and *MT‐TS1*. Sequencing data were compared with the Human Genome Reference (GRCh 37/hg 19) to identify mutant genes and loci. And the GenBank reference sequence of *MARVELD2* is NG_017201.2. Finally, we used online tools such as RDDC (https://rddc.tsinghua‐gd.org/zh), SpliceAI (https://spliceailookup.broadinstitute.org/), and REVEL (https://genome.ucsc.edu/cgi‐bin/hgTrackUi?db=hg19&g=revel) to predict risk from different variants.

## 3 RESULTS

### 3.1 Clinical data

**FIGURE 2:**
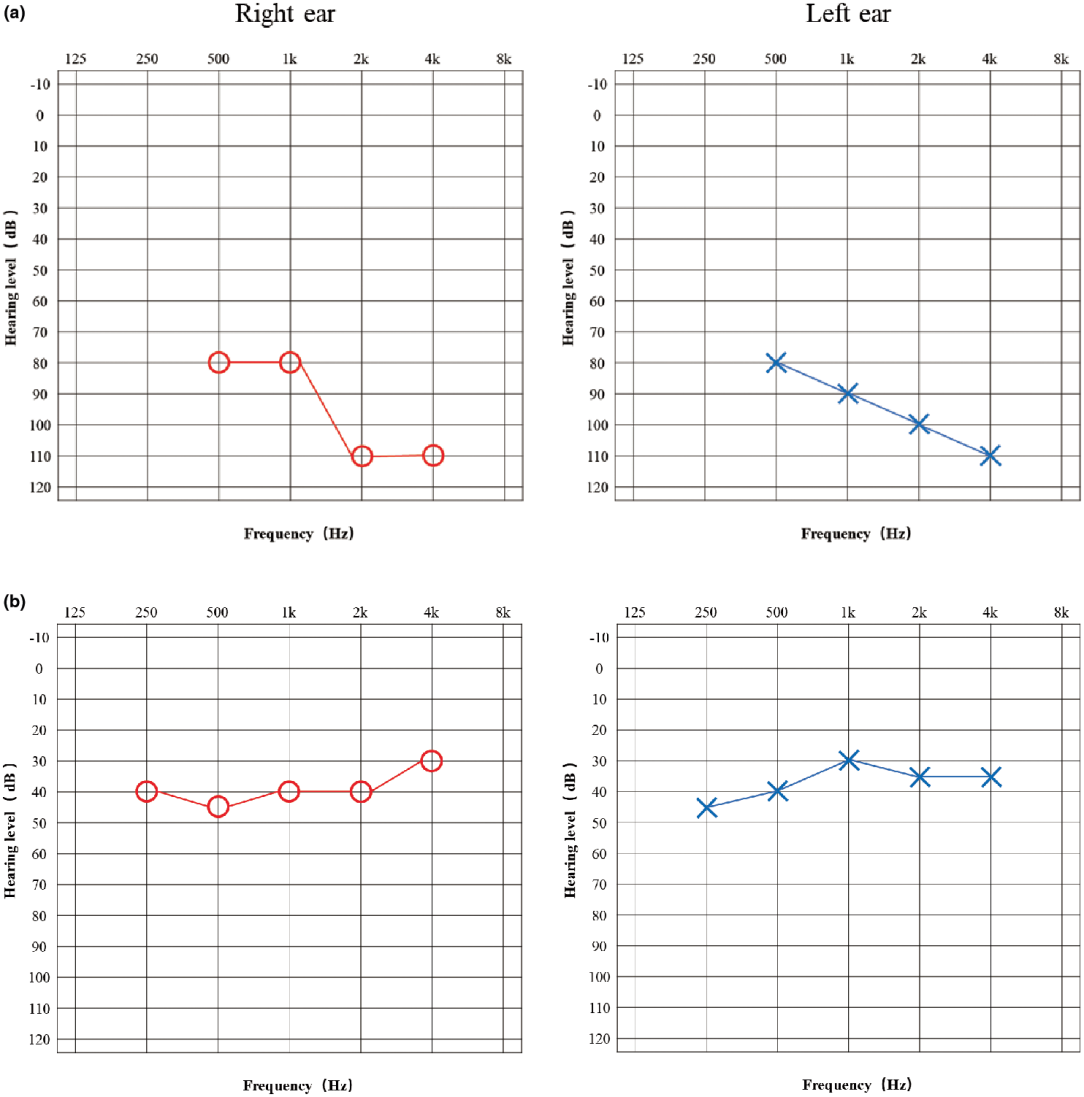
Hearing level of two probands. (a) The result of ASSR of the proband in Family 1 showed the thresholds of her right ear were 80, 80, 110, and 110 dBnHL at 0.5, 1, 2, and 4 kHz, respectively, while the thresholds of her left ear were 80, 90, 100, and 110 dBnHL. (b) The pure tone hearing test showed the hearing thresholds of the Family 2 proband's right ear at 0.25, 0.5, 1, 2, and 4 kHz were 40, 45, 40, 40, and 30 dBnHL, respectively, while the thresholds of his left ear were 45, 40, 30, 45, and 45 dBnHL.

The proband in family 1 failed the newborn hearing screening and was diagnosed with congenital sensorineural hearing loss. The result of ASSR at the age of 2 years and 10 months showed the thresholds of her right ear were 80,80,110 and 110dBnHL at 0.5, 1, 2, and 4 kHz respectively, whereas the thresholds of her left ear were 80, 90, 100, and 110dBnHL (Figure 2a). The ABR showed a binaural response threshold of 90dBnHL. Distortion product otoacoustic emissions (DPOAE) were not elicited at 1, 1.5, 3, 4, and 8 kHz in her right ear and at 0.75, 1, 1.5, 2, 3, and 6 kHz in the left ear.

The proband in family 2 performed a pure tone hearing test at the age of 9 years and 4 months. The hearing thresholds of his left ear at 0.25, 0.5, 1, 2, and 4 kHz were 45, 40, 30, 35, and 35dBnHL respectively, and were 40, 45, 40, 40, and 30dBnHL of right ear (Figure 2b). There was no response to ABR induced by the Click sound, and DPOAE and TEOAE were not elicited in both ears.

### 3.2 Mutation identification data

The genomic DNA sequences of the probands were compared with the human genome reference sequence (GRCh37/hg19).

**FIGURE 3:**
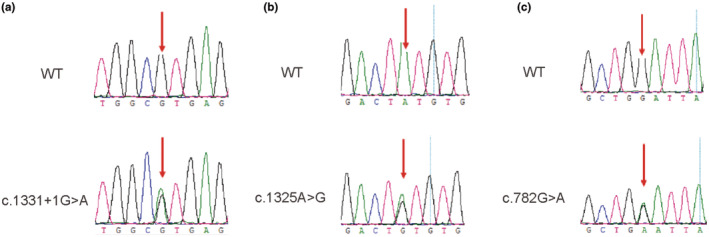
Genetic sequencing results of the probands and their parents. Mutated sequences of the identified (a) c.1331+1G>A, (b) c.1325A>G, and (c) c.782G>A variant and their corresponding wild types. The red arrow indicates the site of the base deletion or substitution.

In family 1, proband II‐1 carries compound heterozygous variant c.1331+1G>A (Figure 3a) and c.1325A>G; p.Tyr442Cys of *MARVELD2* (Figure 3b). The variant c.1331+1G>A was inherited from her father, which is a splice mutation in Intron 4, causing the substitution of no. 1331 + 1 nucleotide from guanine to adenine. According to the gnomAD database, the allele frequency of this variant is 0.00001315. The variant c.1325A>G occurred in Exon 4 of the *MARVELD2* gene, causing the substitution of no.1325 nucleotide from adenine to guanine and of no.442 amino acid from tyrosine to cysteine. There is no record of allele frequency for this variant.

In family 2, proband II‐1 carries compound heterozygous variant c.1331+1G>A and c.782G>A; p.Gly261Glu of *MARVELD2* (Figure 3c). The variant c.1331+1G>A was inherited from her father. The variant c.782G>A occurred in Exon 2 of the *MARVELD2* gene, causing the substitution of no.782 nucleotide from guanine to adenine and of no.261 amino acid from glycine to glutamic acid. The variant was reported to have a minor allele frequency of 0.000006573, whereas the allele frequency in the East Asian population is 0.0001925 in the gnomAD database.

### 3.3 Functional analysis of the mutant protein

*MARVELD2* encodes four transmembrane structural domains and the occludin‐ELL structural domain located at the C‐terminal and is an important protein that makes up the tight junctions between cells.

**FIGURE 4:**
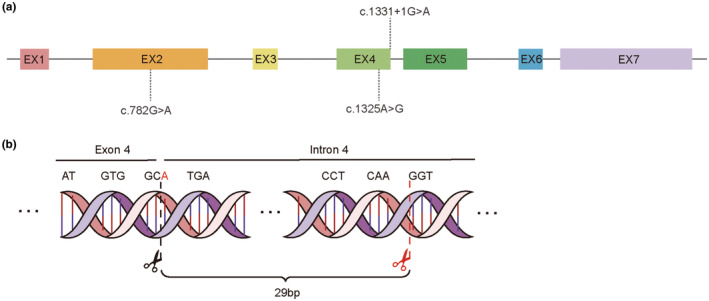
Schematic representation of the MARVELD2 mutation. (a) Sequence positions where the three variants are located. (b) Variant c.1331+1G>A may lead to the emergence of new splice site after the 29th bases in Intron 4 of the hnRNA. Black indicates the wild‐type splice site, and red indicates the new splice site in the predicted results.

Variant c.782G>A is a missense mutation that occurs in exon 2 (Figure 4a), which alters the *MARVELD2* transmembrane structural domains, resulting in a substitution of no. 782 nucleotide from guanine to adenine and of no.261 amino acid from glycine to glutamic acid. There are no reports of pathogenicity in this variant. The probability of this site occurring in a normal population is extremely low. According to the ACMG (American College of Medical Genetics and Genomics)/AMP (Association for Molecular Pathology) guidelines, the evidence supports PM2_Supporting, and therefore this variant was judged to be an uncertain significance mutation. Analysis using the RDDC database suggests that it may be pathogenic (https://rddc.tsinghua‐gd.org/zh).

Variant c.1325A>G is a missense mutation that occurs in exon 4 (Figure 4a), causing the substitution of no.1325 nucleotide from adenine to guanine and of no.442 amino acid from tyrosine to cysteine, and there are no reports of pathogenicity at this locus. Prediction of its protein function using REVEL resulted in a deleterious result. According to the ACMG/AMP guidelines, this variant has evidence of pathogenicity as PM2, and PP3 and is judged to be an uncertain significance mutation. Analysis using the RDDC database suggests that it may be pathogenic (https://rddc.tsinghua‐gd.org/zh).

Variant c.1331+1G>A occurs in the intron 4 (Figure 4a) and results in the substitution of no. 1331 + 1 nucleotide from guanine to adenine. Mutations occurring in the splice junction region may alter the splice position resulting in aberrant splicing of hnRNA affecting subsequent protein translation. We used online biological databases, such as SpliceAI (https://spliceailookup.broadinstitute.org/) and RDDC (https://rddc.tsinghua‐gd.org/zh), to predict whether variant c.1331+1G>A affects splicing and showed that the variant may alter the original receptor site in favor of a new splice site at 1331 + 29, resulting in premature translation termination of the protein (Figure 4c). Based on the ACMG/AMP guidelines, this variant was judged to be a pathogenic mutation with evidence items PVS1, PM2, PM3_Supporting, and PP1_Strong.

## 4 DISCUSSION

None of the two probands in this study passed newborn hearing screening and were diagnosed with congenital sensorineural hearing loss. Their mothers were screened to rule out TORCH (Toxplasma, rubellavirus, Cytomegalo virus, herpes simplex virus and other) infection and had no history of specific medications during pregnancy and perinatally. We screened 218 deafness genes using next‐generation sequencing and Sanger sequencing and found compound heterozygous mutations in *MARVELD2*, and therefore hypothesized that hereditary factors may be the major cause of hearing loss in the probands. However, the fact that the proband in Family 2 exhibited only mild to moderate hearing loss may be related to individual differences, and as animal models exhibiting progressive hearing loss, there may likewise be heterogeneity in the hearing phenotype of patients with *MARVELD2* variants in the population. This patient's hearing should be monitored continuously. In addition, mutations at different sites of MARVELD2 have different effects on protein structure and may have unknown protective factors, which need to be further explored.

The cochlea contains two distinct extracellular fluids, endolymph and perilymph. Although perilymph has an ionic composition similar to that of the general extracellular fluid, endolymph is characterized by a high concentration of K^+^, which is essential for hearing (Wangemann, 2006). The tight junctions between the epithelial cells of the inner ear serve to form an epithelial barrier and separate endolymph and perilymph (Riazuddin et al., 2006). In humans, the longest *MARVELD2* mRNA has seven exons, which are predicted to encode four transmembrane structural domains and the C‐terminal occludin‐ELL structural domain for binding ZO‐1 (Ikenouchi et al., 2005; Riazuddin et al., 2006). Another tight junction protein, ILDR1 (immunoglobulin‐like domain containing receptor 1), does not recruit tricellulin to the cochlear tTJ but rather plays an important role in maintaining the position of tricellulin, and its defects can lead to autosomal recessive hearing loss DFNB42, which further demonstrates the important role of tricellulin in maintaining normal hearing (Morozko et al., 2015). *Marveld2* ^−/−^ mice develop very severe hearing loss early in life and rapidly progress to total deafness, with a large early loss of outer hair cells through apoptosis (Kamitani et al., 2015). *Marveld2* ^R497X/R497X^ mice do not express tricellulin at the tricellular junction of the inner ear sensory epithelium, and this is associated with the absence of tricellulin at the cochlear tTJ. *Marveld2* ^R497X/R497X^ mice, like the deletion mutant mice, show rapidly progressive hearing loss with very severe hearing loss at P30. Mutant mice have changes in the ultrastructure of the tTJ and show loss of cochlear hair cells (Nayak et al., 2013). Disruption of the tight junctions that connect the bicellular and tricellular junctions may affect the paracellular permeability of ions or small molecules, creating a toxic microenvironment for cochlear cells and leading to hearing loss (Kamitani et al., 2015).

As of this writing, 13 *MARVELD2* mutations that may be associated with sensorineural hearing loss have been reported in Pakistani, Slovak, Czech Roma, Hungarian Roma and Chinese families, and 2 other mutations that have not yet been reported were identified in our study (Table 1). Five of these mutations (c.1183‐1G>A, c.1331–1G>A, c.1331+1G>A, c.1331+2T>C, c.1331+2deITGAG) occurred at the splice donor where exon 4 and intron 4 intersect. These mutations may alter the original splice position of hnRNA, and protein translation may be terminated prematurely, resulting in a protein lacking the occludin‐ELL domain encoded by exons 5, 6, and 7 of the *MARVELD2* gene. Exons 4–5 deletion mutation results in the deletion of a large fragment, which also affects the integrity of the occludin‐ELL domain, and may result in the inability of tricellulin to bind to other tight‐junction proteins and to form effective tight junctions. Mutations c.1555delinsAA, and c.1543delA both result in frameshift mutations, where the termination codon appears prematurely and full‐length tricellulin cannot be translated. Seven other missense mutations occur in different structural domains. Mutations c.730G>A, c.772G>A, c.949C>G, and c.1006C>T occur in exon 2, which encodes four transmembrane regions, and the mutations may result in hearing loss due to abnormalities in the protein's structure and function. c.1498C>T occurs in exon 5, and the mutations may likewise result in alterations in occludin‐ELL domain structure and function.

**TABLE 1:** Summary of all pathogenic variants in *MARVELD2.*

Variant	Amino acids substitution	Reference
c.1183‐1G>A		Riazuddin et al. (2006)
c.1331–1G>A		Riazuddin et al. (2006)
c.1331+1G>A		Chishti et al. (2008); Sadeghi et al. (2020), this report
c.1331+2T>C		Riazuddin et al. (2006); Mašindová et al. (2015); Nayak et al. (2015); Šafka Brožková et al. (2012)
c.1331+2deITGAG		Riazuddin et al. (2006)
Exons 4–5 deletion	p.C395‐Q501del	Nayak et al. (2015)
c.1555delinsAA	p.D519Kfs*12	Taghipour‐Sheshdeh et al. (2019)
c.1543delA	p.K517Rfs*16	Babanejad et al. (2012)
c.730G>A	p.G244R	Zheng et al. (2019)
c.772G>A	p.V258M	Zheng et al. (2019)
c.782G>A	p.G261E	This report
c.949C>G	p.R317G	Zheng et al. (2019)
c.1006C>T	p.R336W	Zheng et al. (2019)
c.1325A>G	p.Y442C	This report
c.1498C>T	p.R500X	Riazuddin et al. (2006)

In this study, we report two newly identified *MARVELD2* variants that complement the mutational spectrum of this gene. Although previous cases have mostly focused on Pakistani, Roma, and Iranian populations, recent reports have shown the presence of *MARVELD2* mutations in Chinese populations with different mutation locations, suggesting that other structural domains of tricellulin also play an important role in barrier function formation and hearing maintenance.

## AUTHOR CONTRIBUTIONS

Xinyu Shi and Xiaozhou Liu designed the study. Xinyu Shi, Xiaozhou Liu, Yanjun

Zong and Zhengdong Zhao collected clinical data and predicted the models. Xinyu Shi and Xiaozhou Liu wrote the manuscript. Yu Sun edited the manuscript. And all authors critically reviewed the final version of the manuscript.

## FUNDING INFORMATION

This study was funded by the Innovative Research Groups of Hubei Province (No. 2023AFA038), the National Key Research and Development Program of China (No. 2021YFF0702303, 2023YFE0203200), and the National Natural Science Foundation of China (No. 82071058).

## CONFLICT OF INTEREST STATEMENT

None.

## Supporting information


Table S1.

## Data Availability

The study was approved by Medical Ethics Committee of Tongji Medical College, Huazhong University of Science and Technology. Before participation, all participants or their parents must give written initial informed consent as per the Declaration of Helsinki.
