# Supplementary material for: Novel compound heterozygous variants in MARVELD2 causing autosomal recessive hearing loss in two Chinese families
Source: Mol Genet Genomic Med. 2024 Jul 30;12(8):e2502. doi: 10.1002/mgg3.2502 (PMC11287821; doi:10.1002/mgg3.2502)
Supplement: Supplementary file 1 — Table S1. [file MGG3-12-e2502-s001.docx]

Table S1. 218 genes tested for deafness

| inheritance | Number of genes | Genes tested |
| --- | --- | --- |
| Autosomal recessive nonsyndromic hearing impairment | 72 | *ADCY1,BDP1,BSND,CABP2,CDC14A,CDH23,CIB2,CLDN14,CLDN9,CLIC5,COL11A2,DCDC2,PJVK,ELMOD3,EPS8,EPS8L2,ESPN,ESRP1,ESRRB,FAM65B,GAB1,GIPC3,GJB2,GJB3,GJB6,GRAP,GXCR1,GRXCR2,HGF,ILDR1,KARS1,LHFPL5,LOXHD1,LRTOMT,MARVELD2,MET,MPZL2,MSRB3,MYO15A,MYO3A,MYO7A,NARS2,OTOA,OTOF,OTOG,OTOGL,PCDH15,PDZD7,PNPT1,PPIP5K2,PTPRQ,RDX,ROR1,S1PR2,SERPINB6,SLC26A4,SLC26A5,SPNS2,STRC,SYNE4,TBC1D24,TECTA,TMC1,TMEM132E,TMIE,TMPRSS3,TPRN,TRIOBP,TSPEAR,USH1C,WBP2,WHRN* |
| Autosomal dominant nonsyndromic hearing impairment | 46 | *ABCC1,ACTG1,CCDC50,CD164,CEACAM16,COCH,COL11A1,COL11A2,CRYM,GSDME,DIABLO,DIAPH1,DIAPH3,DMXL2,EYA4,GJB2,GJB3,GJB6,GRHL2,HOMER2,KCNQ4,KITLG,LMX1A,MCM2,MIR96,MYH14,MYH9,MYO6,MYO7A,NLRP3,OSBPL2,P2RX2,PDE1C,PLS1,POU4F3,REST,SIX1,SLC17A8,SLC44A4,TBC1D24,TECTA,TJP2,TMC1,TNC,TRRAP,WFS1* |
| X-link hereditary hearing impairment | 6 | *AIFM1,COL4A6,GPRASP2,POU3F4,PRPS1,SMPX* |
| Y-link hereditary hearing impairment | 1 | *TBL1Y* |
| Maternally inherited hearing impairment | 5 | *MT-RNR1,MT-TS1,MT-TE,MT-TK,MT-TL1* |
| Syndromic hearing impairment | 119 | *ABHD12,ACOX1,ACTG1,ADGRV1,AIFM1,ALMS1,AMMECR1,ARSG,ATP1A3,ATP6V1B1,ATP6V1B2,BCAP31,BCS1L,BMP1,BSND,C10orf2,CACNA1D,CD151,CDH23,CEP250,CEP78,CHD7,CIB2,CISD2,CLPP,CLRN1,COL11A1,COL11A2,COL1A1,COL1A2,COL2A1,COL4A3,COL4A4,COL4A5,COL9A1,COL9A2,COL9A3,COQ6,CRTAP,DLX5,DMXL2,DNAJC3,DNMT1,DSPP,EDN3,EDNRB,ERAL1,EXOSC2,EYA1,FDXR,FGF3,FGFR2,FKBP10,FKBP14,FLNA,FOXI1,GATA3,GFER,GJB2,GPSM2,GRHL2,HARS2,HOXA2,HSD17B4,IARS2,IFITM5,IGF1,KCNE1,KCNJ10,KCNQ1,LARS2,MITF,MYH9,MYO7A,NARS2,NDP,NF2,NLRP3,OPA1,P3H1,PAX3,PCDH15,PDSS1,PDSS2 ,PEX1,PEX6,POLD1,PPIB,PRPS1,SERAC1,SERPINF1,SERPINH1,SIX5,SLC19A2,SLC26A4,SLC33A1,SLC4A11,SLC52A2,SLC52A3,SLC9A1,SLITRK6,SMPX,SNAI2,SOX10,SP7,SPARC,SPATA5,SPTBN4,TBC1D24,TBX1,TCOF1,TIMM8A,TRRAP,TUBB4B,USH1C,USH1G,U SH2A,WFS1,WHRN* |

Note: The number of genes was duplicated across subclasses, with a total of 218 genes detected
